# Supplementary material for: Association between oral microbiome and seven types of cancers in East Asian population: a two-sample Mendelian randomization analysis
Source: Front Mol Biosci. 2023 Nov 21;10:1327893. doi: 10.3389/fmolb.2023.1327893 (PMC10702768; doi:10.3389/fmolb.2023.1327893)
Supplement: Supplementary file 1 [file DataSheet1.ZIP › Supplementary Materials/MR plots of saliva/saliva═╝/Pancreatic cancer/pheno.2595_to_pancreatic cancer_forest.pdf]

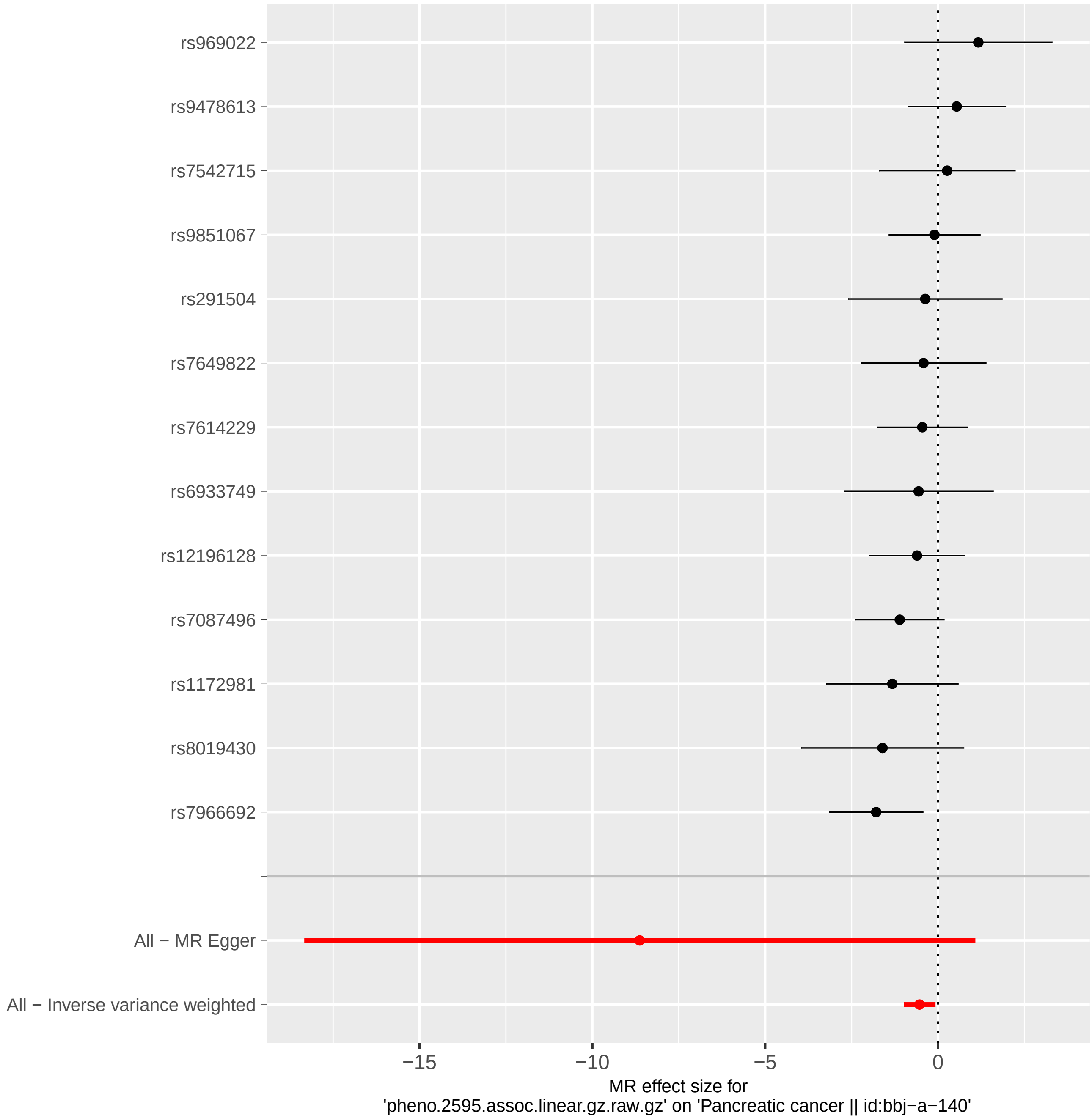

rs969022
rs9478613
rs7542715
rs9851067
rs291504
rs7649822
rs7614229
rs6933749
rs12196128
rs7087496
rs1172981
rs8019430
rs7966692
All − MR Egger
All − Inverse variance weighted
−15
−10
−5
0
MR effect size for
'pheno.2595.assoc.linear.gz.raw.gz' on 'Pancreatic cancer || id:bbj−a−140'
